# 70400 Collaborative Care for Opioid Dependence And Pain (CCODAP): A Pilot Randomized Control Trial of an Opioid Tapering Intervention

**DOI:** 10.1017/cts.2021.504

**Published:** 2021-03-30

**Authors:** Michael Bushey, Kurt Kroenke

**Affiliations:** Indiana University School of Medicine

## Abstract

ABSTRACT IMPACT: If successful, this program can provide a scalable, patient-centered intervention to help patients taper off opioid medications in primary care settings. OBJECTIVES/GOALS: Tapering of chronic opioid therapy is often desirable but challenging in primary care and specialty clinics that lack behavioral health expertise. The objective of this pilot study is to determine the feasibility of testing a peer-delivered pain self-management program to assist primary care patients through an opioid taper. METHODS/STUDY POPULATION: To provide critical support to patients and providers during opioid medication tapering, we propose to conduct a 40 patient randomized controlled pilot of a 12-week telecare collaborative care program administered by a psychiatrist and peer recovery specialist team. The intervention will incorporate a validated positive psychology intervention for treating chronic pain. Additionally, participants will be invited to participate in semi-structured individual interviews to discuss their experience in the trial, what worked well, what could be improved, and potential strategies to bolster recruitment of additional patients in future studies. RESULTS/ANTICIPATED RESULTS: Our primary aim is to determine the effectiveness of our intervention in facilitating opioid medication weaning, with reduction in opioid dose as the primary outcome. Our secondary aims will be to assess pain outcomes, adherence to tapering, patient satisfaction, and barriers to adherence as described by patients. DISCUSSION/SIGNIFICANCE OF FINDINGS: This trial proposes a novel collaborative care approach for opioid weaning using proven, easy-to-deliver positive psychology tools for pain management that, if successful, could be implemented broadly in many clinics struggling to safely reduce opioid prescribing.

